# Prefab Hollow Glass Microsphere-Based Immunosensor with Liquid Crystal Sensitization for Acute Myocardial Infarction Biomarker Detection

**DOI:** 10.3390/bios12070439

**Published:** 2022-06-22

**Authors:** Panpan Niu, Junfeng Jiang, Kun Liu, Shuang Wang, Tianhua Xu, Ziyihui Wang, Tong Wang, Xuezhi Zhang, Zhenyang Ding, Yize Liu, Tiegen Liu

**Affiliations:** 1 School of Precision Instrument and Opto-Electronics Engineering, Tianjin University, Tianjin 300072, China; niupanpan@tju.edu.cn (P.N.); beiyangkl@tju.edu.cn (K.L.); shuangwang@tju.edu.cn (S.W.); xutianhua@tju.edu.cn (T.X.); zyhwang@tju.edu.cn (Z.W.); wangtong1994@tju.edu.cn (T.W.); zhangxz@tju.edu.cn (X.Z.); zyding@tju.edu.cn (Z.D.); lyzdoing@tju.edu.cn (Y.L.); tgliu@tju.edu.cn (T.L.); 2 Key Laboratory of Opto-Electronics Information Technology, Ministry of Education, Tianjin University, Tianjin 300072, China; 3 Key Laboratory of Micro Opto-Electro Mechanical System Technology, Ministry of Education, Tianjin University, Tianjin 300072, China; 4 Tianjin Optical Fiber Sensing Engineering Center, Institute of Optical Fiber Sensing, Tianjin University, Tianjin 300072, China

**Keywords:** biosensors, liquid crystal, whispering gallery mode, biomarkers, cardiac troponin, acute myocardial infarction

## Abstract

Quantitative detection of cardiac troponin biomarkers in blood is an important method for clinical diagnosis of acute myocardial infarction (AMI). In this work, a whispering gallery mode (WGM) microcavity immunosensor based on a prefab hollow glass microsphere (HGMS) with liquid crystal (LC) sensitization was proposed and experimentally demonstrated for label-free cardiac troponin I-C (cTnI-C) complex detection. The proposed fiber-optic immunosensor has a simple structure; the tiny modified HGMS serves as the key sensing element and the microsample reservoir simultaneously. A sensitive LC layer with cTnI-C recognition ability was deposited on the inner wall of the HGMS microcavity. The arrangement of LC molecules is affected by the cTnI-C antigen—antibody binding in the HGMS, and the small change of the surface refractive index caused by the binding can be amplified owing to the birefringence property of LC. Using the annular waveguide of the HGMS, the WGMs were easily excited by the coupling scanning laser with a microfiber, and an all-fiber cTnI-C immunosensor can be achieved by measuring the resonant wavelength shift of the WGM spectrum. Moreover, the dynamic processes of the cTnI-C antigen—antibody binding and unbinding was revealed by monitoring the wavelength shift continuously. The proposed immunosensor with a spherical microcavity can be a cost-effective tool for AMI diagnosis.

## 1. Introduction

Acute myocardial infarction (AMI), the leading cause of death in cardiovascular disease, is triggered by the hypoxia of heart muscle which results in irreversible tissue necrosis [1]. Globally, the rapid and accurate diagnosis of AMI has the potential to save millions of lives, and thus the development of diagnostic methods has sparked a lot of interest in the medical market [2]. Based on the concentration evaluation of AMI biomarkers in blood or saliva, traditional immunoassays with high sensitivity and selectivity, including the enzyme-linked immunosorbent assay (ELISA) [3] and chemiluminescence immunoassay (CLIA) [4], have been developed and applied in hospitals. Among various AMI biomarkers [5,6], cardiac troponins (cTn), including cTnI and cTnT, are the preferred options for clinical diagnosis because of their high sensitivity and specificity for myocardial necrosis [7]. In addition, recent studies have shown that the evaluation of the cTn level can help in the early medication and risk stratification of COVID-19 patients [8].

However, traditional immunoassays relied on by professional laboratories present some challenges, such as the large amount of sample consumption, the complicated operation, and long time for each test [9], which limit the rapid and low-cost diagnosis of AMI with time-related survival. In addition to traditional immunoassays, the convenient use of biosensors to assess the cTn concentration has become one of the most expectant diagnostic tools for AMI prediction [10]. At present, electrochemical (ELC) [11], field-effect transistor (FET) [12], lateral flow immunoassay (LFIA) [13], and optical fiber [14,15,16,17,18] biosensors are attractive for point-of-care testing (POCT) [19], which facilitate a whole new horizon for possible treatment and prevention of cardiovascular diseases. With the development of materials and manufacturing technologies, optical fiber biosensors in particular have been exploited for cTn assays in recent years owing to their fiber waveguide structure and biocompatible silica materials. Wang, et al. proposed a localized surface plasmon resonance (LSPR) optical fiber biosensor assisted with 2D nanomaterials for cTnI measurement [14]. The fiber surface was functionalized with an enzyme to improve the selectivity performance of the biosensor, and a restricted limit of detection (LOD) of 96.2638 ng/mL was obtained. Microfibers are often used for biosensors due to their strong surface evanescent fields for biomolecular detections [20]. Zhou, et al. utilized the interference turning effect of the optical microfiber coupler for ultrasensitive detection of cTnI [15], but the ultrahigh sensitivity was only achieved within a small range of 10 fg. For grating-based biosensors, Liu, et al. exploited a phase-shifted microfiber grating for label-free cTnI detection [16]. The spectroscopy-based biosensor provides a fine reflective spectrum for detection resolution improvement. Ran, et al. reported an evanescent field biosensor based on harmonic resonances microfiber grating [17], and the impact of the thermal noise could be reduced by the harmonic resonances with different responses. In addition, the chemiluminescent optical fiber cTnI biosensor with a unique all-directional chemiluminescent collection vial for sensitivity improvement was also reported [18], and its LOD is as low as 0.31 pg/mL. Although some optical fiber biosensors have been explored for cTnI detection, the sensing elements of fiber sensors with lengths of several millimeters to centimeters need to be immersed in the samples to be tested, and the consumptions of samples and reagents still cannot meet the requirements of microanalysis. The fiber biosensors lacking inherent microcavity structures require additional microfluidic packages [21,22], resulting in increased cost of use. Furthermore, functional materials [23,24] that take advantage of the high refractive index (RI) sensitivity of optical fiber need to be further explored for detecting sensitization and signal amplification.

Taking the stable binary complex of cTnI as the target biomarker, a fiber-optic immunosensor based on a prefab hollow glass microsphere (HGMS) with liquid crystal (LC) modification for high-sensitivity cTnI-C detection was proposed in this work. The proposed cTnI-C immunosensor has an optical whispering gallery mode (WGM) microcavity composed of a perforated HGMS, which acts as both the sensing element and the sample reservoir for microanalysis. The LC molecules on the surface layer were vertically oriented by the induced surfactant, and the perpendicular alignment of the LC molecules will be disturbed by the binding of the cTnI-C antigen—antibody in the microcavity. By utilizing the birefringent property of LC, the cTnI-C concentration-dependent binding was transformed into the effective RI change of the HGMS, and the quantitative detection of the biomarker cTnI-C can be realized by tracking the resonant wavelength shift of the WGM excited on the HGMS. The optical immunosensor demonstrated here has the ability to monitor the process of biomolecular reaction with low sample consumption, which provides a prospective all-fiber scheme for the diagnosis of AMI and the microanalyses of biomarkers.

## 2. Materials and Methods

### 2.1. Materials and Reagents

Standard communication single-mode fiber (SMF, Catalog No. SMF-28e) was fabricated by Corning Inc. (New York, NY, USA). The hollow glass microsphere (HGMS, Catalog No. K25) was fabricated by 3M Company (Maplewood, MN, USA). Sulfuric acid (98 wt.%, Catalog No. 0090) was purchased from Jiangtian Chemical Technology Co., Ltd. (Tianjin, China). Hydrogen peroxide (30 wt.%, Catalog No. A97384) was purchased from Innochem Sciences & Technology Co., Ltd. (Beijing, China). Anhydrous ethanol (Catalog No. E809056) was purchased from Macklin Biochemical Co., Ltd. (Shanghai, China). Glutaraldehyde (GA, Catalog No. G105906) and (3-aminopropyl)triethoxysilane (APTES, Catalog No. A107148) were purchased from Aladdin Biochemical Technology Co., Ltd. (Shanghai, China). Dimethyloctadecyl [3-(trimethoxysilyl)propyl] ammonium chloride (DMOPA, Catalog No. A-FF032) and nematic LC 4′-pentyl-4-cyanobiphenyl (5CB, Catalog No. C486A) were purchased from Xianding Biotechnology Co., Ltd. (Shanghai, China). Glycine (Catalog No. G8200), bovine serum albumin (BSA, Catalog No. A8020), goat anti-rabbit IgG (Catalog No. SPA134), recombinant human C-reactive protein (CRP, Catalog No. CLP1016), and sterile deionized water (Catalog No. F0020) were purchased from Solarbio Science and Technology Co., Ltd. (Beijing, China). Protein elution buffer (0.1 M pH3.5 glycine-HCl buffer, Catalog No. MP005M) was purchased from M&C Gene Technology Ltd. (Beijing, China). Human prostate specific antigen (PSA, Catalog No. CSB-DP274I) was purchased from Huamei Biotech Co., Ltd. (Wuhan, China). Native human serum albumin (ALB, Catalog No. bs-0945P), FITC-conjugated monoclonal mouse anti-cardiac troponin I antibody (Catalog No. V3201-FITC), recombinant human cardiac troponin I-C (cTnI-C) complex protein (Catalog No. bs-41212P), and phosphate-buffered saline (PBS) solution (0.01M pH 7.2~7.4, Catalog No. C01-01001) were purchased from Biosynthesis Biotechnology Inc. (Beijing, China). Piranha solution was prepared by mixing sulfuric acid and hydrogen peroxide with volume ratio of 7:3 for 30 min. The cTnI-C samples were prepared by diluting the cTnI-C complex protein with the 40 mg/mL ALB-PBS solution in different concentrations.

### 2.2. Fabrication and Principle of the Immunosensor

**Figure 1:**
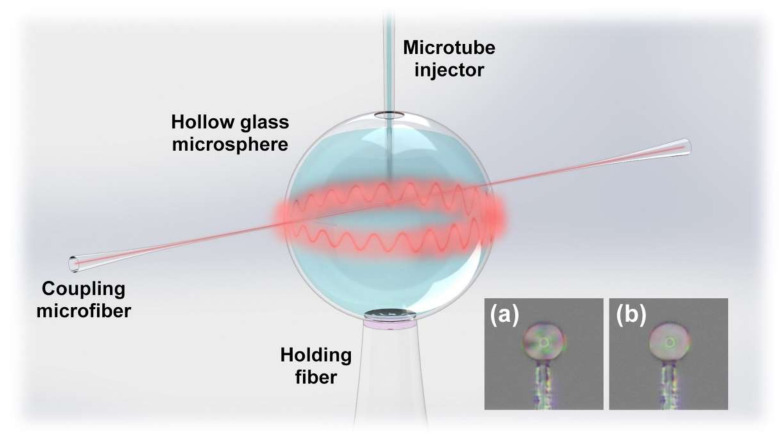
Schematic diagram of the immunosensor based on the prefab HGMS for AMI biomarker detection. Inset: Polarized optical images of the HGMS filled with LC in (**a**) anisotropic and (**b**) isotropic phases.

**Figure 2:**
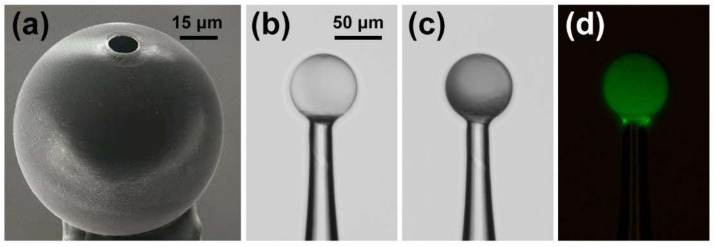
(**a**) SEM image of the perforated HGMS with a 10 μm diameter microhole; (**b**) Optical microscopic images of the HGMS (**b**) before and (**c**) after LC modification; (**d**) Fluorescence microscopic image of the HGMS immobilized FITC-cTnI antibody.

**Figure 3:**
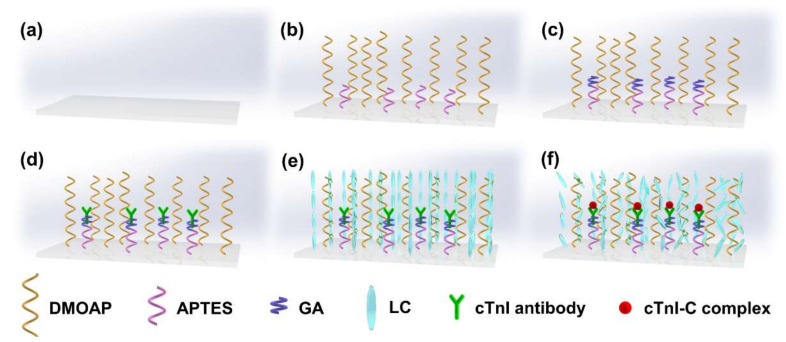
Schematic illustration of the inner wall functionalization and detection strategy of the HGMS. (**a**) bare inner wall; (**b**) silanization; (**c**) aldehyde modification; (**d**) antibody incubation; (**e**) LC modification; (**f**) antigen capture.

The proposed fiber-optic immunosensor has a simple structure, which is composed of the HGMS, holding fiber, and coupling microfiber, as shown in Figure 1. The manufacturing process of the immunosensor is illustrated in Supplementary Figure S1. The HGMS as the key sensing element was adhered on a SMF tip, and then perforated by focused ion beam-scanning electron microscope (FIB-SEM, ZEISS Crossbeam 540) to punch a microhole for LC sensitization and the cTnI-C sample injection. Figure 2a illustrates the SEM image of the perforated HGMS with a 10 μm diameter microhole. The more economical perforating methods can be realized by using a steel needle or tapered capillary, as shown in Supplementary Figure S2. The modification reagents and cTnI-C samples were injected into the perforated HGMS (Supplementary Figure S3) through a microtube injector assisted with a digital syringe pump, as shown in Supplementary Figure S4. The soda lime borosilicate HGMSs, which also serve as the microsample reservoirs, have small diameters of 25~105 μm and thin wall thicknesses less than 1 μm; hence, the consumptions of the filled samples are as low as 0.06~4.85 nL. Figure 3 illustrates the inner wall functionalization and detection strategy of the HGMS. For the specific capture of the cTnI-C antigen, the antibody anchoring procedure based on LC modification was performed as follows:

1. Hydroxylation: Inject piranha solution into the HGMS for 30 min and then wash with anhydrous ethanol and deionized water.
2. Silanization (Figure 3b): Inject the mixed solution of 1% (*v*/*v*) APTES and 1% (*v*/*v*) DMOPA into the hydroxylated HGMS for 30 min, wash with anhydrous ethanol and deionized water, and dry at 110 °C for 30 min.
3. Aldehyde modification (Figure 3c): Inject 2% (*v*/*v*) GA solution into the silanized HGMS for 30 min, and then wash with anhydrous ethanol and deionized water.
4. Antibody incubation and blocking (Figure 3d): 20 μg/mL FITC-cTnI antibody PBS solution was injected into the aldehyde-modified HGMS for 60 min, washed with PBS buffer, and then injected with 80 mM glycine solution for 60 min to block the aldehyde site of the unbound antibody, washed with deionized water.
5. LC modification (Figure 3e): Inject 5CB LC after heating to >35 °C into the HGMS for 60 min, and finally clean with deionized water.

Figure 2b,c illustrates the optical microscopic images of the HGMS before and after LC modification. The LC coating on the inner wall of the HGMS reduced the light transmittance, and furthermore, the anchoring of the FITC-conjugated cTnI antibodies was confirmed by the fluorescence microscopic image (characterized by DSZ2000X, UOP) with green fluorescence emission. On the self-assembly LC-modified layer, the surfactant DMOPA can induce the homeotropic arrangement of LC molecules, and APTES with amino groups can bind with the bifunctional cross-linking GA for the cTnI antibody anchoring. When the sample was injected into the LC modification HGMS, the cTnI-C antigens were captured by the cTnI antibody anchored on the inner wall of the HGMS (Figure 3f), and then the homeotropic alignment of the LC molecules was disturbed [25]. With the increase of antigen—antibody binding, the LC molecules were gradually transformed from the anisotropic phase (Inset of Figure 1a) to the isotropic phase (Inset of Figure 1b), and the effective RI of the LC layer was correspondingly changed from extraordinary RI (*n*_e_ = 1.67) to ordinary RI (*n*_o_ = 1.51) [26,27]. Therefore, the weak surface RI variation caused by biomarker cTnI-C binding was amplified owing to the birefringence property of LC, which can be sensed by the RI-sensitive optical WGM propagating along the circumferential direction of the HGMS.

To excite the WGM in the circumferential direction of the HGMS, the interrogation scanning laser (Agilent 81607A) was coupled into the HGMS through a <3 μm diameter microfiber prepared by the hydroxide flame taper method [28], as shown in Supplementary Figure S5. The WGM coupled from the HGMS was detected by an optical power sensor (Agilent 81636B + 8164B) synchronized with the scanning laser. As an equivalent WGM resonator, the HGMS with microfiber can provide RI-sensitive WGM under the resonant condition [29]:

(1) $$2\pi n_{eff}R=m\lambda_{m}$$

where n_eff_ is the effective RI of HGMS, R is the radius of HGMS, m is the number of modes, λ_m_ is the resonant wavelength. The free spectral range (FSR) of the resonant spectrum can be expressed as:

(2) $$FSR=\frac{\lambda_{m}^{2}}{2\pi n_{eff}^{m}R}$$

**Figure 4:**
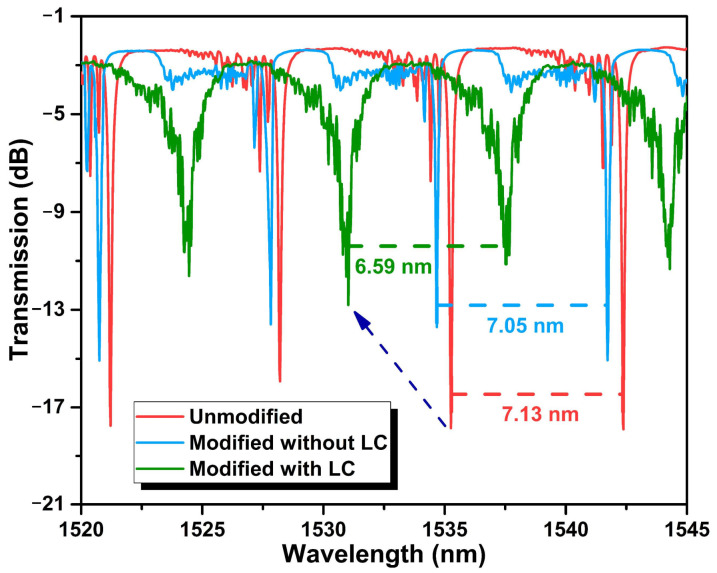
WGM spectra of the unmodified and modified HGMSs.

The variable FSRs were verified by exciting WGMs in HGMSs with different diameters, as shown in Supplementary Figure S6. Figure 4 shows the WGM spectra of the unmodified and modified HGMSs. Due to the thin wall of the HGMS and the high coupling efficiency of the microfiber, the WGM on the unmodified HGMS with a high Q factor of 1.94 × 10^4^ and a FWHM of 0.08 nm can be obtained as shown in Supplementary Figure S7. After LC modification, the effective RI of the HGMS was increased and the FSR was reduced, and the LC modification changed the flatness and uniformity of the HGMS surface, causing a spectral degradation and a reduced Q factor of 2.63 × 10^3^ as shown in Supplementary Figure S8 (Appendix A) [30]. According to Equation (1), the LC-sensitization cTnI-C detection in the HGMS microcavity can be realized by tracking the resonant wavelength of the WGM spectrum, on account of the birefringence of the LC layer.

## 3. Results and Discussion

### 3.1. Static Response to cTnI-C Complex

**Figure 5:**
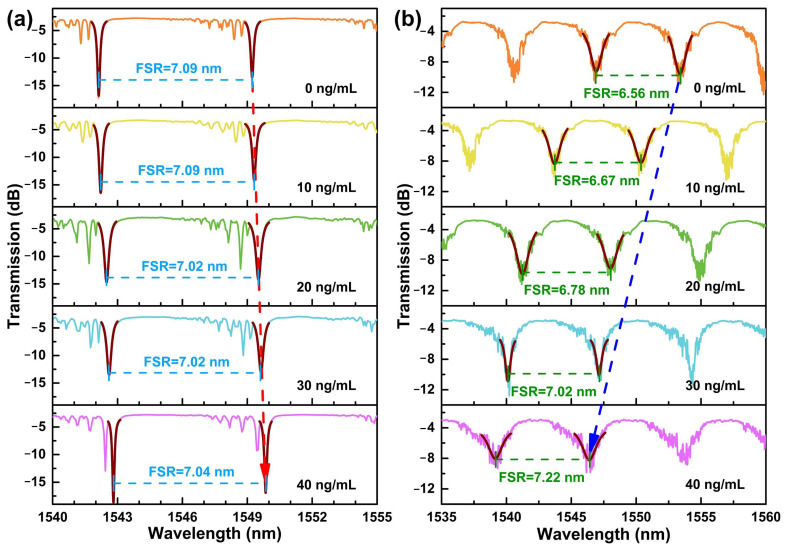
WGM spectral evolutions of (**a**) the non-LC modified and (**b**) the LC modified HGMSs with increasing cTnI-C concentration. The red and blue arrows indicate the redshift and blueshift of the resonant wavelengths.

**Figure 6:**
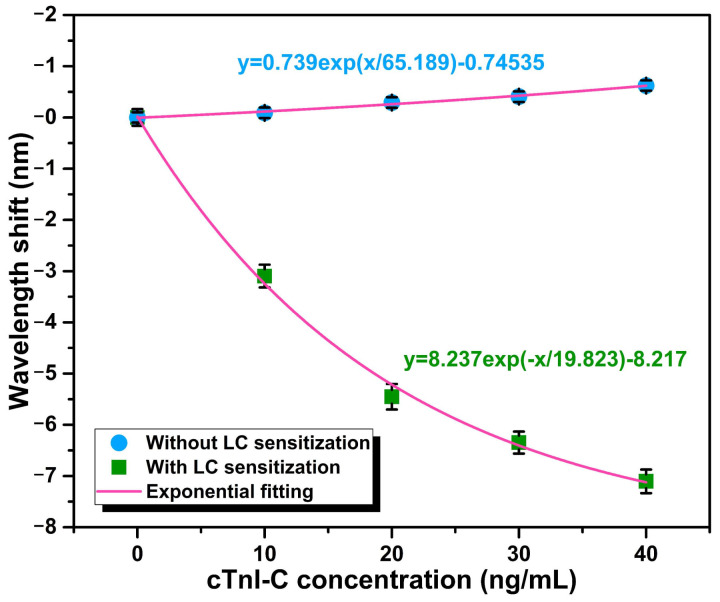
Relationship between the resonant wavelength shifts and the cTnI-C concentration.

During the static response test, the cTnI-C samples ranging from 0 to 40 ng/mL with a step of 10 ng/mL were individually injected into the HGMS, and the WGM spectra at different concentrations were recorded for 10 times after 30 min of the cTnI-C samples injection. After each sample test is completed, the waste sample in the HGMS was extracted by the microtube injector, and the mild protein elution buffer was employed for test initialization [15,31]. Furthermore, in order to verify the LC sensitization for cTnI-C detection, the same HGMS with LC sensitization was initialized with piranha solution after completing the static response test, and then implemented antibody incubation again without LC modification for comparison. Figure 5a,b shows the WGM spectral evolutions of the non-LC modified and the LC modified HGMSs with increasing cTnI-C concentration. The notches of the resonant spectra were depicted by Lorentz fitting for accurate tracking. As the cTnI-C concentration increases from 0 to 40 ng/mL, more antigen—antibody binding events occurred on the inner wall surface, the effective RI of HGMS increased, and there is a consequent redshift in the resonant wavelength. Due to the weak RI variation, the FSR hardly changes. Different from the non-LC modified HGMS, the effective RI of the HGMS decreases because the nematic LC layer induced by DMOAP was disturbed (the effective RI of the birefringence LC layer was changed from *n*_e_ = 1.67 to *n*_o_ = 1.51), and the resonant notches of WGM spectra drift to shorter wavelengths with the increasing cTnI-C concentration. The relationship between the resonant wavelength shifts and the cTnI-C concentration is plotted in Figure 6. During the measurement range of 0~40 ng/mL, the static responses were represented by using exponential fitting, and the LC-modified HGMS has more wavelength shifts than the HGMS without LC sensitization. The weak RI change caused by biomarker cTnI-C binding was amplified by the sensitive LC layer with the birefringence property and the comparison of FSR changes in Supplementary Figure S9 also verified the sensitization effect of the LC layer for cTnI-C detection.

### 3.2. Dynamic Response to cTnI-C Complex

**Figure 7:**
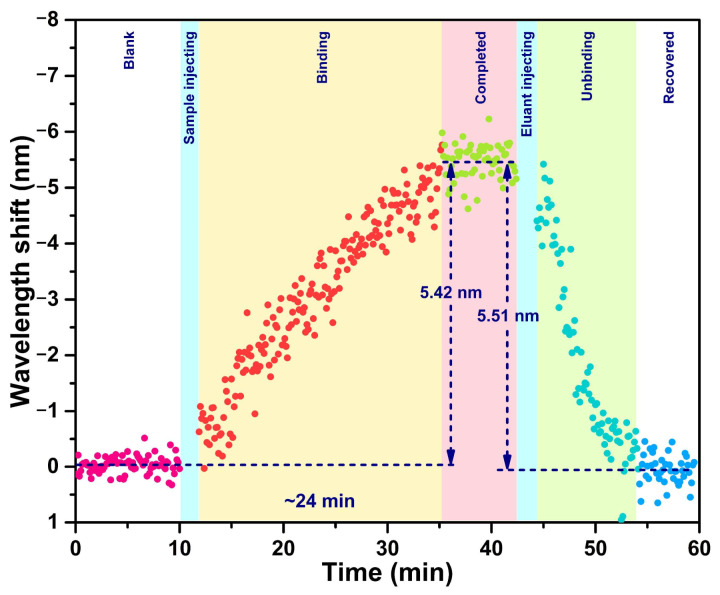
Dynamic response of the resonant wavelength shift of the HGMS with LC sensitization to the binding and unbinding processes of the cTnI-C antigen—antibody from 0 to 20 ng/mL. The different colored data points represent the resonant wavelengths at different detection stages.

**Figure 8:**
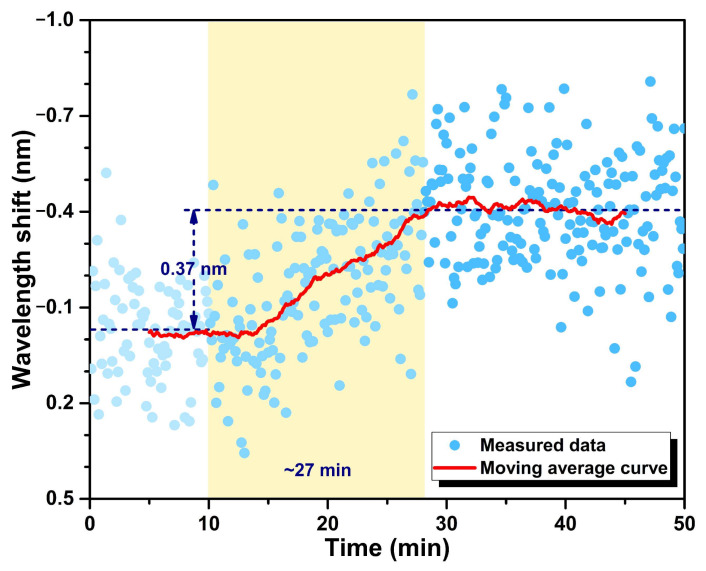
Dynamic response of the resonant wavelength shift of the HGMS with LC sensitization to the low concentration variation from 0 to 1 ng/mL.

Thanks to the all-fiber detection scheme, the proposed fiber-optic immunosensor has the ability to monitor the biomolecular reaction on the optical microcavity surface in real time. The dynamic response processes of the cTnI-C antigen—antibody binding and unbinding were demonstrated by recording the resonant wavelength shift of the LC sensitization HGMS consecutively, as shown in Figure 7. After 20 ng/mL cTnI-C sample injecting, the resonant wavelength shift at 0 ng/mL progressively increases with a response time of ~24 min, and subsequently recovered by elution with antigen—antibody unbinding; the wavelength shifts of binding and unbinding are 5.42 and 5.51 nm, respectively. In addition, the dynamic response of the wavelength shift to the low concentration variation was also tested. According to the static response curve in Figure 6, the LOD of the proposed immunosensor can be calculated as 1.103 ng/mL (Appendix B) [32]; hence, the low concentration of 1 ng/mL was selected for validation. Figure 8 shows the dynamic response of the resonant wavelength shift of the HGMS with LC sensitization to the low concentration variation from 0 ng/mL to 1 ng/mL. For direct observation, the resonant wavelength shifts at different times were processed by using the moving average algorithm [33]. It can be seen from the result curve that the low concentration variation of 1 ng/mL with response time of ~27 min, a wavelength shift of 0.37 nm was easily detected owing to the ingenious LC sensitization. In addition, a comprehensive comparison between the proposed LC-sensitization and the reported cTnI immunosensors is given in Table S1. Compared with our previous works [34,35,36], the proposed immunosensor with LC sensitization avoids additional gain configuration and complex demodulation, provides comparable LOD and resolution with ultralow consumption, making it more suitable for the microanalysis of biomarkers.

### 3.3. Stability and Specificity

**Figure 9:**
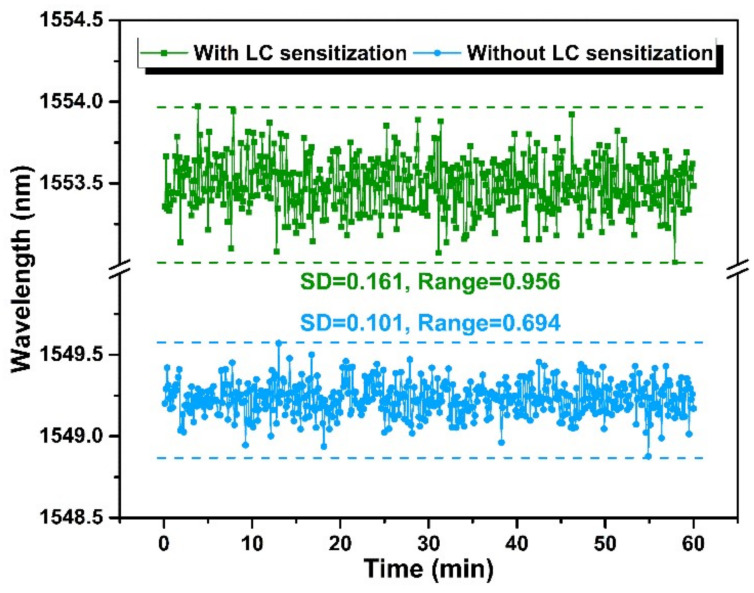
A comparison of the HGMSs with and without LC sensitization in resonant wavelength stability at the cTnI-C concentration of 0 ng/mL.

To assess the stability of the immunosensor, the resonant wavelengths of the HGMSs with and without LC sensitization were surveyed during 60 min at the cTnI-C concentration of 0 ng/mL, and the statistical results are shown in Figure 9. The standard deviation (SD) and the range of the resonant wavelength shift of the HGMS with LC sensitization is larger than that of the HGMS without LC sensitization, which indicates that the LC sensitization partly reduces the stability of the sensor, but the higher sensitivity obtained is worthwhile for high-sensitivity biomarker detection.

**Figure 10:**
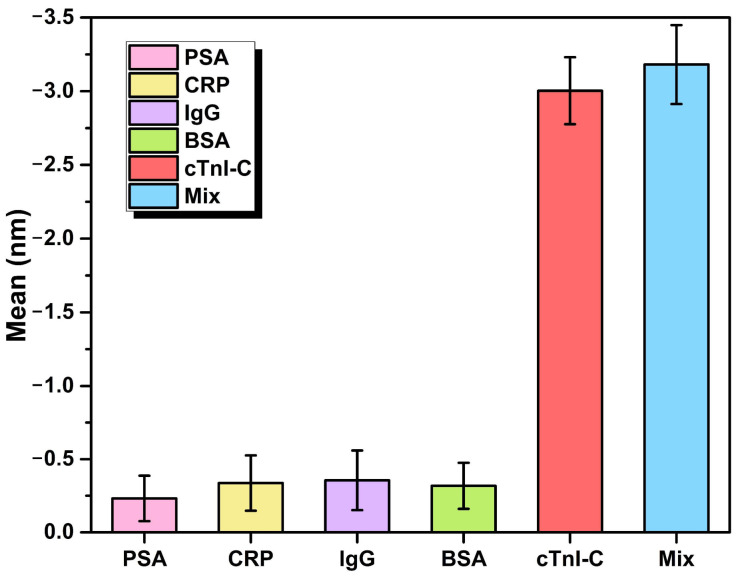
Resonant wavelength shifts of the HGMS with LC sensitization to the interferences and cTnI-C samples with a same concentration of 10 ng/mL for specificity test.

With the exception of stability, the specificity is also important for biomarker detection in various biomolecules. The specificity of the proposed LC sensitization immunosensor was assessed by using the four kinds of non-specific interference samples: PSA, CRP, IgG, and BSA. Each sample was injected into the HGMS at the same concentration of 10 ng/mL in 40 mg/mL ALB-PBS solution to simulate the complex measurement conditions of human serum. Figure 10 shows the resonant wavelength shifts of the HGMS with LC sensitization to the interferences and cTnI-C samples. The resonant wavelength has more significant drifts to the samples containing cTnI-C, demonstrating the good specificity and selectivity of the immunosensor for cTnI-C detection.

## 4. Conclusions

Overall, a novel WGM microcavity immunosensor based on the LC-sensitized HGMS for myocardial biomarker cTnI-C detection was proposed and demonstrated in this work. The WGM with a high Q factor of 1.94 × 10^4^ was excited on the HGMS by using a coupling microfiber. The tiny HGMS with an injection microhole serves as the sensing element and the microsample reservoir simultaneously. A self-assembly LC-modified layer was built on the inner wall of the HGMS microcavity for sensitization, and the LC molecules were initially induced to be arranged in an ordered space by DMOAP. With the increase of cTnI-C antigen—antibody binding events, the order of LC molecules with birefringence property was disturbed, resulting in significant effective RI changes from extraordinary to ordinary RIs. Therefore, LC-amplified cTnI-C biomarker detection of the sample in the HGMS microcavity can be realized by measuring the resonant wavelength shift of the WGM spectrum. Experimental results show that the LOD of the immunosensor is 1.103 ng/mL during a range of 0~40 ng/mL, the sensitization effect of LC molecules was verified by comparing with the non-LC modified sensor, and the spectra of the LC-modified and non-LC modified sensor have the opposite wavelength shift directions because of the different RI change mechanisms. Due to the high sensitivity improved by LC molecules, the developed immunosensor has the ability to monitor the dynamic binding and unbinding processes of the cTnI-C antigen—antibody. In addition, the low concentration variation in the LOD level was revealed by moving average. Our fiber-optic immunosensor with prefab microcavity has the advantages of a miniature size, high sensitivity, low LOD, and low sample consumption, which provides a cost-effective solution for label-free and real-time biomarker detection in cardiovascular diseases diagnoses.

## Data Availability

Not applicable.

## Supplementary Materials

The following supporting information can be downloaded at: https://www.mdpi.com/article/10.3390/bios12070439/s1, Figure S1: illustration of the manufacturing process of the immunosensor; Figure S2: economical perforating methods by using (a) stainless steel needle or (b) tapered hard capillary; (c,d) SEM images of the HGMSs perforated by economical methods; Figure S3: optical microscopic images of the HGMS with different filling states; Figure S4: microtube injector assisted with a digital syringe pump; Figure S5: optical microscopic images of the HGMS coupled with microfiber in (a) side and (b) top views, (c) indicated with red-laser; (d) Photo of the coupling platform for HGMS microcavity with microfiber; Figure S6: (a) WGM spectra with different FSRs in different diameters HGMSs; (b) relationship between the FSR and the diameter of HGMS; Figure S7: spectrum of the WGM on unmodified HGMS with a high Q factor of 1.94 × 10^4^ and a FWHM of 0.08 nm; Figure S8: spectrum of the WGM on LC-modified HGMS with a Q factor of 2.63 × 10^4^ and a FWHM of 0.59 nm; Figure S9: FSR changes of the HGMSs with and without LC sensitization in different cTnI-C concentrations; Table S1: A comprehensive comparison between the proposed LC-sensitization and the reported cTnI immunosensors.

## Appendix A. Calculation of Q Factor

The Q factor is an important indicator for evaluating the optical WGM resonators, which is defined by the time-averaged energy in the cavity divided by the energy loss per cycle. For the WGM sensing spectrum, the full width at half maximum (FWHM) of the resonant notch directly determines the detection limit of the WGM sensor, and the WGM sensors with a narrow FWHM can detect small refractive index changes or particle adsorption. The expression of the Q factor can be simplified as [30]:

(A1) $$Q=\frac{\lambda_{\mathrm{res}}}{\mathrm{FWHM}}$$

where λres is the resonant wavelength of WGM spectrum.

In the manuscript, the high Q factor of 1.94 × 10^4^ was obtained from the spectrum data of the unmodified HGMS. According to Equation (A1), the Q factor of the WGM on the unmodified HGMS with a FWHM of 0.08 nm at the resonant wavelength of 1554.87 nm can be calculated as 1.94 × 10^4^. In addition, the LC modification changed the flatness and uniformity of the HGMS surface, causing a spectral degradation. In order to track resonant wavelength, the notches of the resonant spectra were depicted by Lorentz fitting during the biomarker detection, as shown in Supplementary Figure S8. Considering a broadened FWHM of 0.59 nm, the Q factor of the modified HGMS with LC at the resonant wavelength of 1550.93 nm was estimated as 2.63 × 10^3^.

## Appendix B. Calculation of LOD

The limit of detection (LOD) indicates one of the most crucial performance parameters for biosensors. According to the International Union of Pure and Applied Chemistry (IUPAC) recommendation, the LOD can be calculated by [32]:

(A2) $$\mathrm{LOD}=f^{-1}({\bar{y}}_{\mathrm{blank}}+3\sigma_{\max})$$

where *f* ^−1^ is the inverse function of the fitted response curve of the biosensor, ${\bar{y}}_{\mathrm{blank}}$ is the mean value of the repeated measurements of the blank sample, $\sigma_{\max}$ is the maximum standard deviation obtained among all the experimental points. By using the fitted exponential curve in Figure 6 as follow:

(A3) f=8.237exp(−x/19.823)−8.217

The inverse function of *f* can be obtained as:

(A4) $$f^{-1}=[19823\times \log((1000\times ({\bar{y}}_{\mathrm{blank}}+3\sigma_{\max}))/8237+8217/8237)]/1000$$

where ${\bar{y}}_{\mathrm{blank}}$ = 0 and $\sigma_{\max}$ = 0.1637. Therefore, the LOD of the proposed WGM immunosensor could be calculated as 1.103 ng/mL.
