# Supplementary material for: Prefab Hollow Glass Microsphere-Based Immunosensor with Liquid Crystal Sensitization for Acute Myocardial Infarction Biomarker Detection
Source: Biosensors (Basel). 2022 Jun 22;12(7):439. doi: 10.3390/bios12070439 (PMC9312929; doi:10.3390/bios12070439)
Supplement: Supplementary file 1 [file biosensors-12-00439-s001.zip › biosensors-1779664-supplementary.pdf]

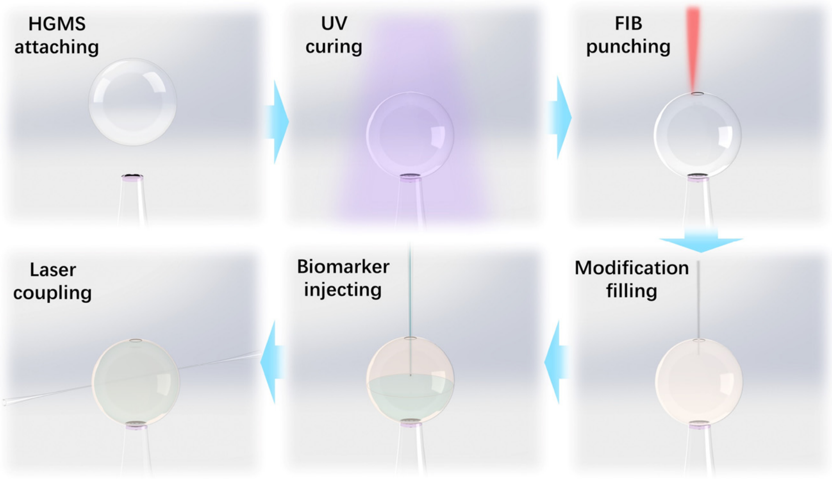

Figure S1. Illustration of the manufacturing process of the immunosensor.

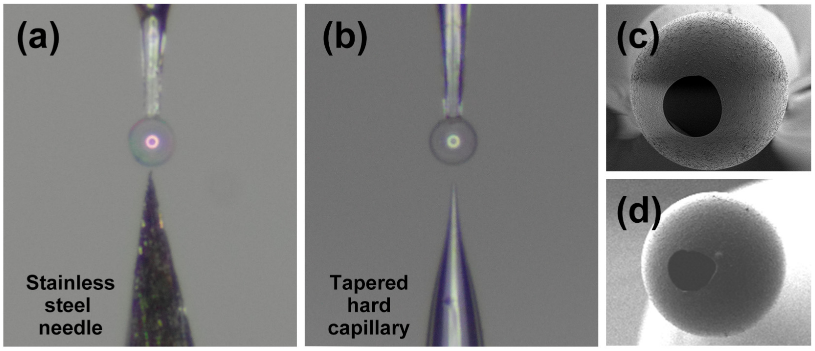

Figure S2. Economical perforating methods by using (**a**) stainless steel needle or (**b**) tapered hard capillary. (**c**,**d**) SEM images of the HGMSs perforated by economical methods.

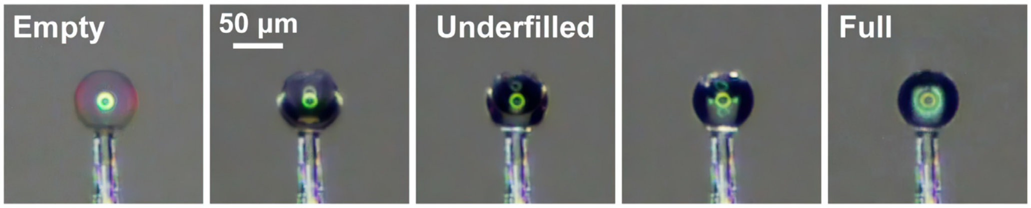

Figure S3. Optical microscopic images of the HGMS with different filling states.

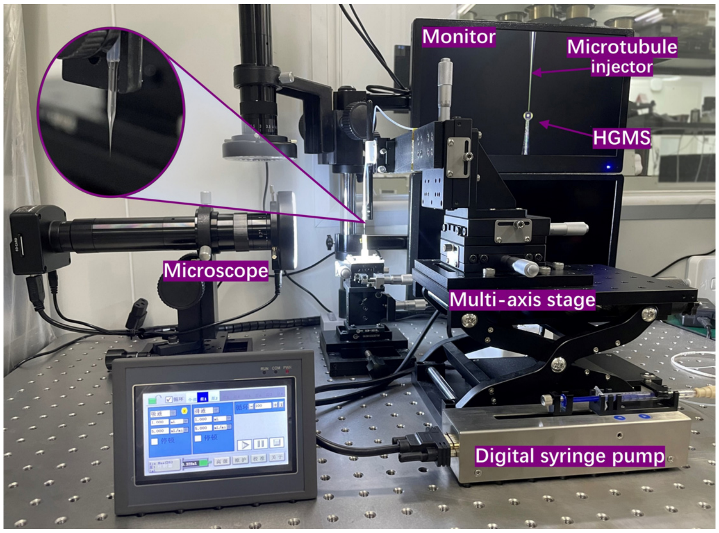

Figure S4. Microtube injector assisted with digital syringe pump.

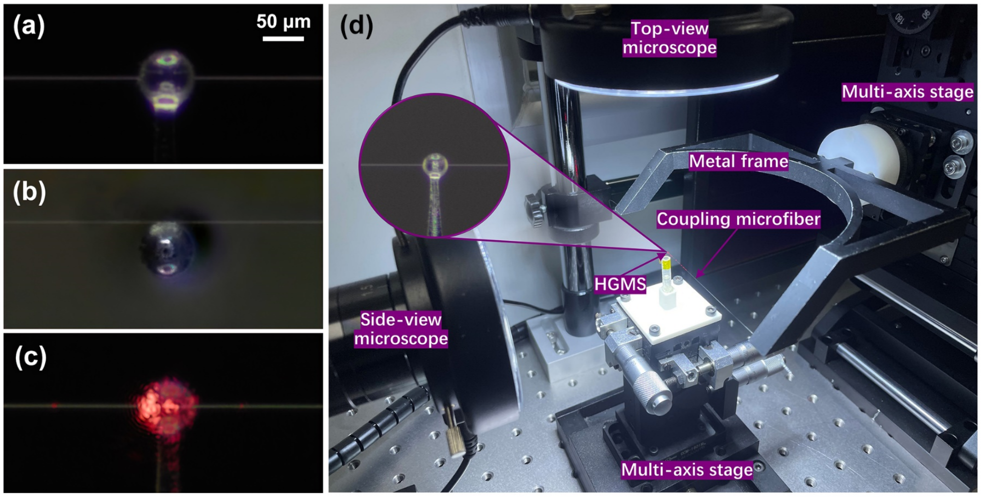

Figure S5. Optical microscopic images of the HGMS coupled with microfiber in (**a**) side and (**b**) top views, (**c**) indicated with red-laser. (**d**) Photo of the coupling platform for the HGMS microcavity with microfiber.

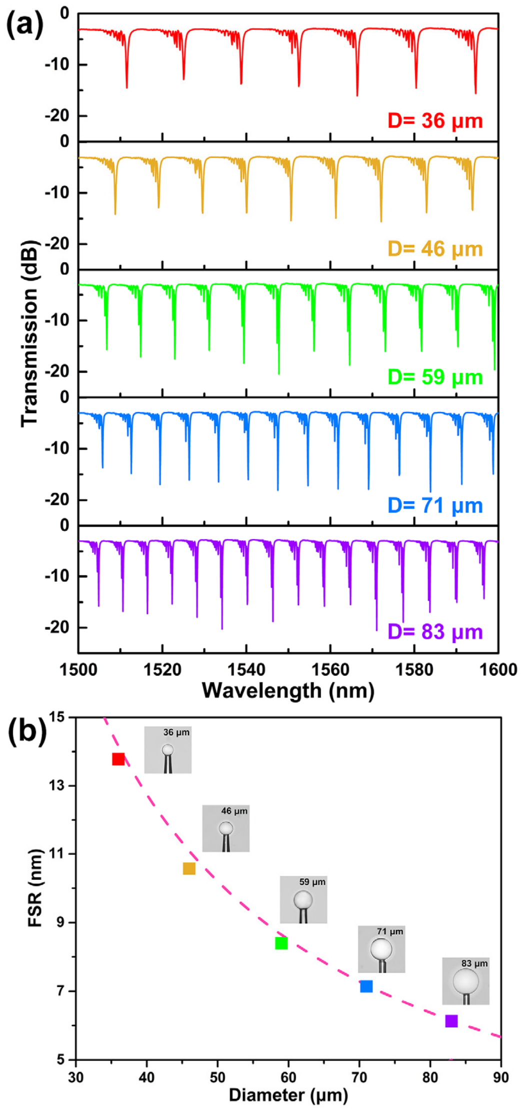

Figure S6. (**a**) WGM spectra with different FSRs in different diameters HGMSs. (**b**) Relationship between the FSR and the diameter of HGMS.

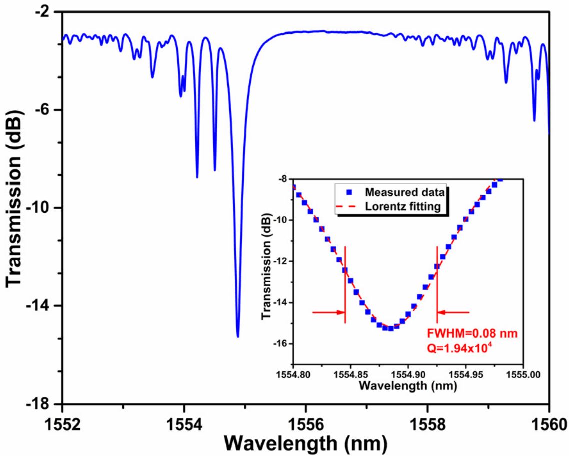

Figure S7. Spectrum of the WGM on the unmodified HGMS with a high Q factor of 1.94×10^4 and a FWHM of 0.08 nm.

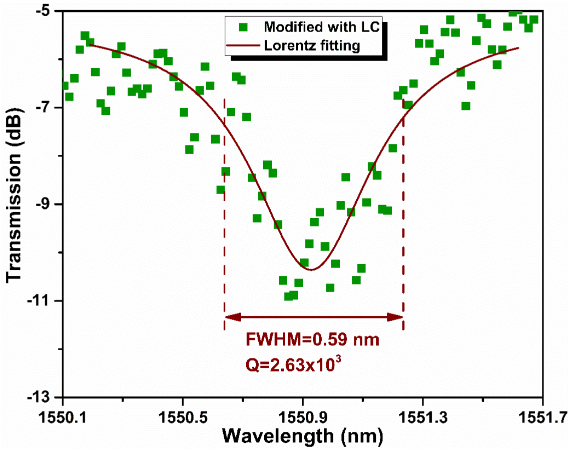

Figure S8. Spectrum of the WGM on the LC-modified HGMS with a Q factor of $2.63×10^4$ and a FWHM of 0.59 nm.

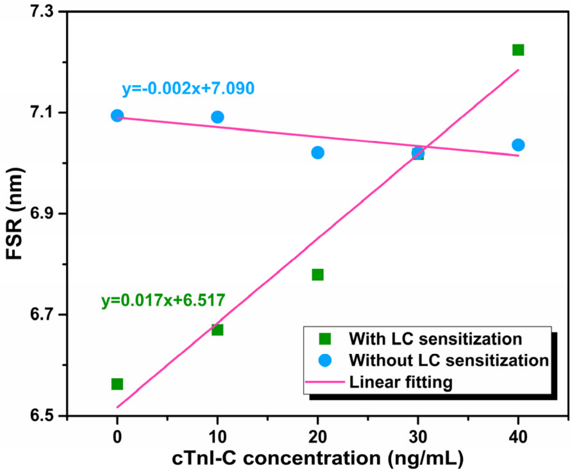

Figure S9. FSR changes of the HGMSs with and without LC sensitization in different cTnI-C concentrations.

Table S1. A comprehensive comparison between the proposed LC-sensitization and the reported cTnI immunosensors.

Sensing element	Sensing element size (mm)	Sensitivity (nm/(ng/mL))	Resolution** (ng/mL)	LOD (ng/mL)	Concentration range (ng/mL)	System configuration	Features	Ref.
LSPR multimode fiber	>1000	$3.4×10^{-3}$	0.29	96.26	0~1000	Simple	Nanomaterials assist	[14]
Optical microfiber coupler	>1000	900*	$1.11×10^{-6*}$	$2×10^{-3}$	0~0.01	Simple	Ultrahigh sensitivity	[15]
Phase-shifted	3	0.1*	0.01*	0.03	0~100	Simple	Reflection-	[16]

mFBG							type	
Harmonic mFBG	4	0.01*	0.1*	13.5	0~1000	Simple	Temperature compensation	[17]
Microtubule	~2000	6.759	1.48×10-4	0.137	0~0.55	Complex	Microfluidic tube, optofluidic lasing	[34,35]
WGM probe	1.2	1.637	6.11×10-4	0.59	0~2.0	Complex	ultrahigh resolution	[36]
LC-modified HGMS	~0.07	0.175	5.71×10-3	1.103	0~40	Simple	Ultralow consumption	This work

*The calculation results were estimated according to the optimal data in the references.

**At the same optical wavelength resolution of 1 pm.
